# Expression of the breast cancer resistance protein and 5*-*fluorouracil resistance in clinical breast cancer tissue specimens

**DOI:** 10.3892/mco.2013.143

**Published:** 2013-07-04

**Authors:** MIN WANG, XIANMING WANG, JIANHUI YUAN, LIANGFENG GUO

**Affiliations:** 1 Department of Breast Surgery, The Second People’s Hospital of Shenzhen, P.R. China; 2 Shenzhen Center for Disease Control and Prevention, Shenzhen, Guangdong 518035, P.R. China

**Keywords:** breast cancer resistance protein, 5*-*fluorouracil, breast cancer, resistance

## Abstract

The breast cancer resistance protein (BCRP) is a recently characterized xenobiotic half*-*transporter protein that acts as an energy*-*dependent efflux pump and may be associated with the multidrug*-*resistant phenotype. The aim of this study was to determine the association between BCRP expression and 5*-*fluorouracil (5*-*FU) resistance in clinical breast cancer tissue specimens. The BCRP expression was investigated using quantitative reverse*-*transcriptase polymerase chain reaction (RT*-*PCR) by use of the Master SYBR-Green I reagent and immunohistochemistry (IHC) by use of the BXP*-*21 anti*-*BCRP monoclonal antibody in clinical breast cancer tissue specimens. Chemosensitivity to 5*-*FU for BCRP*-*positive clinical breast cancer tissue specimens was colorimetrically assessed with the cytotoxicity assay through methyl thiazolyl tetrazolium (MTT) reduction. A total of 37 BCRP*-*positive clinical breast cancer tissue specimens were identified with quantitative RT*-*PCR and IHC. There was a significant correlation in BCRP expression between the results of quantitative RT*-*PCR and IHC in the specimens. The fold resistance to 5*-*FU was 7–12 compared to sensitivity to paclitaxel as determined by the colorimetric assay through MTT reduction in the 37 specimens. Our study results indicated that 5*-*FU resistance may be mediated by BCRP expression in clinical breast cancer tissue specimens, which may help optimize the design of breast cancer clinical chemotherapy schemes in BCRP*-*positive specimens.

## Introduction

Multidrug resistance (MDR) is a major obstacle to successful cancer chemotherapy, including breast cancer. Expression of plasma membrane ATP*-*binding cassette (ABC) transporter proteins that act as efflux pumps to actively extrude drug molecules out of the cell is one of the predominant mechanisms of MDR (1,2). P*-*glycoprotein (P*-*gp), the first drug resistance ABC transporter to be identified (3), has been under extensive investigation for >15 years as a mediator of MDR. Since then, there has been a rapid increase in the number of identified ABC transporter proteins. The multidrug resistance-associated protein was the second ABC transporter protein to be identified (4), followed by several other MRP family members.

The breast cancer resistance protein (BCRP) is a recently characterized xenobiotic half*-*transporter protein that was first identified in the MCF-7/AdrVp breast cancer cell line, which has a multidrug*-*resistant phenotype, notwithstanding the addition of a P*-*gp-blocking agent (verapamil, Vp) (5,6). BCRP is a newly identified member of the ABC protein family and acts as an energy*-*dependent efflux pump (7,8).

BCRP has already been closely investigated. Previous studies indicated that BCRP confers an atypical MDR phenotype (9–11). The established transfectant BCRP-expressing cell lines share a characteristically high resistance to the anthracenedione mitoxantrone, anthracyclines such as daunorubicin and doxorubicin, topotecan, bisantrene and SN*-*38, the active form of irinotecan, whereas they maintain sensitivity to cisplatin, paclitaxel and vinca alkaloids such as vincristine and vinblastine (12). However, the drug*-*resistance spectrum and the mechanisms of action of BCRP have not been fully elucidated.

A transfectant BCRP expression cell model was established (13) and utilized to screen clinical anticancer drugs *in vitro*. Our previous study results demonstrated that resistance to 5*-*fluorouracil (5*-*FU) may be particularly mediated by conjugation with BCRP, which acts as a drug extrusion pump in the cell model (14). Moreover, cell resistance to 5*-*FU*-*induced apoptosis may be reinforced by BCRP expression (15). 5*-*FU is currently one of the most widely used anticancer agents due to its strong anticancer activity. Our previous study demonstrated resistance to 5*-*FU in clinical breast cancer cells: ~2.5% of clinical breast cancer cells exhibited low*-*degree sensitivity and 20% exhibited moderate sensitivity to 5*-*FU (16). In addition, BCRP expression was reported in 20–30% of clinical breast cancer tissue specimens (17).

Whether BCRP expression is involved in clinical breast cancer resistance to 5*-*FU has not been elucidated. It was hypothesized that BCRP expression is positive in clinical breast cancer tissue exhibiting low sensitivity to 5*-*FU. In the present study, BCRP expression was assessed in clinical breast cancer tissue specimens using quantitative reverse*-*transcriptase polymerase chain reaction (RT*-*PCR) by use of the Master SYBR-Green I reagent and immunohistochemistry (IHC) by use of the BXP*-*21 anti*-*BCRP monoclonal antibody. In addition, chemosensitivity to 5*-*FU for the BCRP*-*positive specimens was colorimetrically assessed with the cytotoxicity assay through methyl thiazolyl tetrazolium (MTT) reduction. The aim of this study was to further elucidate the association between BCRP expression and 5*-*FU resistance in clinical breast cancer tissue specimens and optimize breast cancer clinical chemotherapy schemes in BCRP*-*positive specimens.

## Materials and methods

### Tissue specimens

A total of 37 clinical breast cancer tissue specimens from female patients aged 25–50 years were obtained at the time of excision at the Deparment of General Surgery of the Second People’s Hospital of Shenzhen. The placental tissue of a 27-year-old puerperant was obtained from the Department of Obstetrics of the Second People’s Hospital of Shenzhen and was used as a positive control for BCRP expression in quantitative RT*-*PCR and IHC.

### Total RNA isolation and quantitative RT-PCR

Total RNA was extracted from the clinical breast cancer tissue specimens and the placental tissue using the TRIzol reagent (Invitrogen Life Technologies, Carlsbad, CA, USA) according to the manufacturer’s protocol. Staining of the ribosomal bands on 1% agarose gel with ethidium bromide was used to assess the quality of the isolated RNA. The RNA (1 μg) was reverse transcribed with reverse transcriptase using random hexamers as primers (Promega, Madinson, WI, USA). The resulting complementary DNAs (cDNAs) were diluted by 2.5*-*fold with DNase/RNase*-*free water and the levels of BCRP and the housekeeping gene β*-*actin were determined by quantitative PCR on the Mx4000 LightCycler (Stratagene, La Jolla, CA, USA) using the Master SYBR-Green I reagent kit under the following conditions: a step of denaturation at 95°C for 10 min and 40 cycles at 95°C for 5 sec, at 55°C for 30 sec and at 72°C for 30 sec. The endpoint used in PCR quantification (Ct) was defined as the PCR cycle number that crosses an arbitrarily placed signal threshold and is a function of the amount of target cDNA present in the starting material. The BCRP levels were normalized to those of β*-*actin in the same samples. The primers used were 5′*-*tccact gctgtggcattaaa*-*3′ and 5′*-*tgctgaaacactggttggtc*-*3′ for BCRP and 5′*-*tccgtggagaagagctacga*-*3′ and 5′*-*gtacttgcgctcagaaggag*-*3′ for β*-*actin.

### IHC

IHC was performed on the frozen sections of clinical breast cancer and placental tissue that were formalin-fixed and paraffin-embedded. Briefly, the sections were deparaffinized, rehydrated and then blocked for endogenous peroxidase. Following antigen retrieval using a standard microwave method, the sections were incubated in 10% (v/v) normal goat serum to block non-specific binding, followed by overnight incubation with the BXP*-*21 mouse anti*-*human BCRP monoclonal antibody (Alexis Biochemicals, San Diego, CA, USA) at 4°C and at a dilution of 1:50. For the negative control, phosphate-buffered saline was used instead of the primary antibody. Subsequently, a horseradish peroxidase*-*conjugated rabbit anti*-*mouse secondary IgG antibody (Dako, Carpinteria, CA, USA) was used at 1:100 dilution for 1 h at room temperature. The labeling was visualized using 0.05% (w/v) chromogen 3,3′-diaminobenzidine containing 0.01% (v/v) H_2_O_2_ in 0.05 M Tris/HCl buffer (pH 7.6) until a brown reaction was observed by microscopy. The reaction was arrested by immersion in distilled water. The sections were counterstained with haematoxylin and were then dehydrated in ethanol, cleared in xylene, mounted onto glass slides and examined under a light microscope. For semiquantification of the immunostaining, section staining was performed in triplicate sets to confirm the results. The intensity of immunostaining for BCRP was semiquantitatively scored as follows: −, absent; +, weak; ++, moderate; +++, strong.

### Cytotoxicity assay

Clinical breast cancer tissue samples were dissociated into single-cell suspensions, as previously described (9,18,19). Cells were placed in 96-well plates (1–3×10^5^ cells/well) and were exposed to various concentrations of 5*-*FU and paclitaxel for 3 days at 37°C in a humidified atmosphere containing 5% CO_2_. Cell viability was colorimetrically assayed through MTT reduction. Absorbance (570 nm) was determined. Cytotoxicity was expressed as the IC_50_, defined as the drug concentration leading to 50% growth inhibition. The fold resistance (FR) to 5*-*FU was estimated from the ratio of the IC_50_ for 5*-*FU in the cells compared to the IC_50_ for paclitaxel.

### Data analysis

For each experiment, each concentration was tested in triplicate. Each experiment was repeated 2–4 times and found to be reproducible. For statistical analyses, SPSS 13.0 software for Windows was used (SPSS Inc., Chicago, IL, USA). Error bars are presented as standard errors of the mean. To identify statistically significant differences among the means, one*-*way ANOVA was performed. P<0.01 was considered to indicate a statistically significant difference.

## Results

### BCRP mRNA expression in clinical breast cancer tissue specimens

To detect BCRP expression at the mRNA level in clinical breast cancer tissue specimens, high-quality total RNA was extracted from breast cancer and control placental tissue. Subsequently, the Ct value of BCRP and the housekeeping gene β*-*actin were determined by quantitative RT*-*PCR by use of the Master SYBR-Green I reagent. Our results demonstrated that 37 clinical breast cancer tissue specimens expressed BCRP at different levels. The BCRP levels were normalized to those of β*-*actin in the same samples (Table I). The rate of BCRP expression in clinical breast cancer tissue specimens was 26% (37/140). Our results were consistent with those previously reported by Kanzaki *et al*(20), which suggested that BCRP expression may contribute to the failure of breast cancer chemotherapy to a certain extent.

### BCRP protein expression in clinical breast cancer tissue specimens

**Figure 1:**
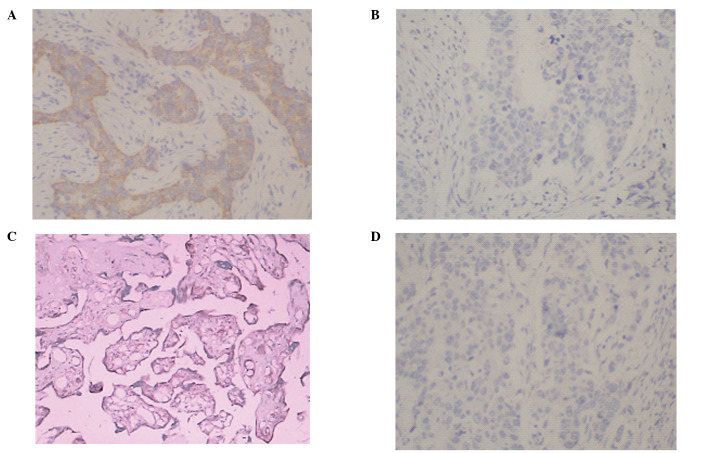
Immunohistochemical staining patterns with BXP-21 mouse monoclonal antibody against breast cancer resistance protein (BCRP) in clinical tissue specimens. Expression of BCRP in frozen tissue sections of clinical breast cancer and placental tissue specimens. A horseradish peroxidase-conjugated rabbit anti-mouse IgG secondary antibody was used. Color development was performed using the chromogen 3,3′-diaminobenzidine. Cells were counterstained with haematoxylin and examined under a light microscope at a magnification of ×100. (A) Representative staining pattern of BCRP-positive and (B) BCRP-negative expression in tumor cell membranes. (C) Representative staining pattern of BCRP-positive expression in control placental tissue cell membranes. (D) Representative staining pattern of blank control for BCRP expression (the primary BXP-21 antibody was replaced with phosphate-buffered saline).

To confirm the previously observed BCRP mRNA expression, IHC assay was performed on the frozen sections of clinical breast cancer and placental tissue by use of the BXP*-*21 anti*-*BCRP monoclonal antibody and the positive ratio of BCRP expression was 22% (28/140). There was no significant difference in BCRP expression between the results of PCR and IHC in the specimens (P>0.05). Our results demonstrated that the expression of BCRP at the protein level was reflected by a corresponding increase in the mRNA level for clinical breast cancer tissue specimens. The representative staining pattern observed in clinical specimens and control placental tissue are shown in Fig. 1.

### Chemosensitivity of BCRP-positive clinical breast cancer tissue specimens to 5-FU

**Figure 2:**
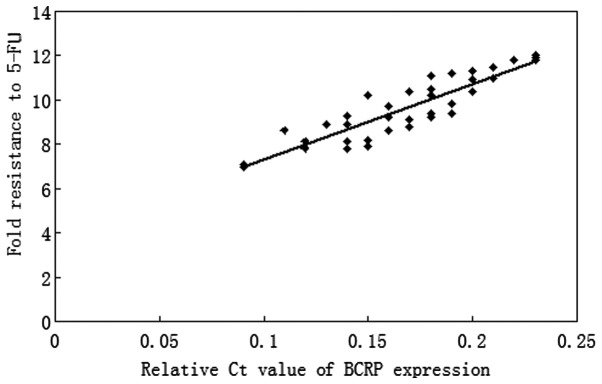
Correlation between breast cancer resistance protein (BCRP) expression and 5-fluorouracil (5-FU) resistance in 37 clinical breast cancer tissue specimens. The relative Ct value of BCRP was determined by quantitative reverse-transcriptase polymerase chain reaction. BCRP expression levels were normalized to those of β-actin in the same samples. The fold resistance to 5-FU was estimated from the ratio of the IC_50_ for 5-FU in the cells compared to the IC_50_ for paclitaxel. Data are representative of at least three independent experiments. There was a statistical correlation between BCRP expression and 5-FU resistance in the 37 clinical breast cancer tissue specimens. Correlation coefficient (R^2^)=0.8124, P<0.01.

To investigate the association of BCRP with 5*-*FU sensitivity, 37 BCRP*-*positive clinical breast cancer tissue specimens were identified with quantitative RT*-*PCR and IHC. There was a significant correlation in BCRP expression between the results of quantitative RT*-*PCR and IHC in those specimens. Subsequently, chemosensitivity of the BCRP*-*positive clinical specimens to 5*-*FU was colorimetrically assessed with the cytotoxicity assay through MTT reduction. Cytotoxicity was expressed as the IC_50_, defined as the drug concentration leading to 50% growth inhibition. The FR to 5*-*FU was estimated from the ratio of the IC_50_ for 5*-*FU in the cells compared to the IC_50_ for paclitaxel. Our results demonstrated that the FR to 5*-*FU was 7–12 compared to the sensitivity to paclitaxel in those 37 specimens. A linear correlation was observed between BCRP*-*positive expression and 5*-*FU resistance (Fig. 2).

## Discussion

BCRP is a recently described ABC half-transporter that confers resistance to certain cancer chemotherapeutic drugs. The drug*-*resistant spectrum and mechanisms of action of BCRP are under constant investigation, mainly focusing on its clinical association with MDR.

Results from previous studies demonstrated that BCRP may mediate resistance to 5*-*FU in the cell model (19,20). 5*-*FU is currently used as a front-line anticancer agent due to its strong anticancer activity. A previous study reported that 22.5% of clinical breast cancer cells exhibit resistance to 5*-*FU (21). In addition, BCRP expression has been reported in 20–30% of clinical breast cancer tissue using the RT*-*PCR assay (22). Whether BCRP expression confers clinical breast cancer resistance to 5*-*FU has not been elucidated.

In this study, we investigated whether BCRP expression in clinical breast cancer is involved in resistance to 5*-*FU. Quantitative RT*-*PCR was employed to rapidly assess BCRP expression in clinical breast cancer tissue specimens. BCRP mRNA was previously detected by quantitative RT*-*PCR in certain drug*-*resistant cell lines (23). Our results demonstrated that the rate of BCRP expression was 26% (37/140) in clinical breast cancer tissue specimens. Our data were consistent with those previously reported by Kanzaki *et al*(19). Subsequently, IHC with the BXP*-*21 anti*-*BCRP monoclonal antibody was used to further confirm BCRP expression in clinical BCRP*-*positive breast cancer tissue. The IHC assay demonstrated a BCRP expression rate of 22% (28/140) in tumor cell membranes, which may be attributed to the threshold of IHC detection. Our results lead to the conclusion that the immunostaining of the cell membranes with BXP*-*21 monoclonal anti-BCRP antibody is highly associated with a marked expression of BCRP mRNA and also suggest that quantitative RT*-*PCR may be extensively used to detect gene expression.

There has been extensive investigation focusing on the failure of 5*-*FU chemotherapy. Our previous study results demonstrated that cell resistance to 5*-*FU*-*induced apoptosis is possibly reinforced by BCRP expression *in vitro*(17). To confirm the associaton between BCRP expression and 5*-*FU resistance in clinical samples, the chemosensitivity for 37 BCRP*-*positive clinical breast cancer tissue specimens to 5*-*FU was determined by quantitative RT*-*PCR and colorimetrically assessed with the cytotoxicity assay through MTT reduction. The results demonstrated that the FR to 5*-*FU was 7–12 in those 37 specimens with different levels of BCRP expression. The BCRP expression was highly correlated with 5*-*FU resistance in the BCRP*-*positive clinical breast cancer samples. However, the complete mechanisms that underlie BCRP-mediated 5*-*FU resistance, such as the functional BCRP domain that is linked to 5*-*FU resistance, have not been fully elucidated.

In conclusion, our study results further confirmed that 5*-*FU resistance may be mediated by BCRP expression in clinical breast cancer tissue specimens and may provide evidence supporting the role of BCRP as a mediator of 5*-*FU resistance, which may help optimize breast cancer clinical chemotherapy schemes in BCRP*-*positive specimens.

## Figures and Tables

**Table I:** BCRP expression measured in clinical breast cancer and placental tissue specimens with quantitative RT-PCR.

	Specimens		
	
Variables	Positive BCRP expression	Negative BCRP expression	Placenta^c^
Relative Ct value^a^	0.16±0.07	-	0.45±0.05
No. of specimens	37	103	1
% Ratio^b^	26 (37/140)	74 (103/140)	-

a Relative Ct value is the value calculated as the mean ± SE of breast cancer resistance protein (BCRP) expression levels normalized to those of β-actin in the clinical samples with quantitative reverse-transcriptase polymerase chain reaction (RT-PCR) and is representative of at least two different experiments.

b %Ratio is the percentage of positive or negative BCRP expression in the clinical samples.

c The relative Ct value of placental specimens is representative of a high BCRP expression.

BCRP, breast cancer resistance protein; -, no BCRP expression.
